# Neural Network‐Based Permittivity Engineering of Magnetic Absorbers for Customizable Microwave Absorption

**DOI:** 10.1002/advs.202521945

**Published:** 2026-01-22

**Authors:** Chenxi Liu, Jinzhe Li, Sen Li, Zhongqiu Guo, Yao Chen, Tian Li, Renchi Qin, Jiaxu Sun, Yongxi Lu, Fanbin Meng

**Affiliations:** ^1^ Key Laboratory of Advanced Technologies of Materials (Ministry of Education) School of Materials Science and Engineering Southwest Jiaotong University Chengdu China; ^2^ Shanghai Key Laboratory of Digital Manufacture For Thin‐Walled Structure Shanghai Jiao Tong University Shanghai China; ^3^ Shanghai Key Laboratory of Materials Laser Processing and Modification School of Materials Science and Engineering Shanghai Jiao Tong University Shanghai China; ^4^ Chongqing CEPREI Industrial Technology Research Institute Co., Ltd Chongqing China

**Keywords:** microwave absorption, magnetic composites, neural network, permittivity, ultrathin absorber

## Abstract

The development of high‐performance microwave absorbers featuring ultrathin profiles and customizable bandwidth remains a formidable obstacle for cutting‐edge electromagnetic stealth and long‐term service applications. Traditional design approaches for absorbers often rely on inefficient trial‐and‐error methods, a challenge exacerbated in magnetic absorbers by the intricate coupling between permittivity and permeability. This work introduces a neural network‐based permittivity engineering strategy, underpinned by a novel “permeability locking‐permittivity optimization” paradigm that effectively decouples the interdependent electromagnetic parameters. A high‐throughput permittivity feature space was constructed via tensor‐based electromagnetic theory calculations, and a dual‐task screening strategy was implemented to identify optimal and effective absorption conditions. This data‐driven framework facilitated the inverse design of a magnetic composite, culminating in the guided synthesis of flaky carbonyl iron/barium titanate composites. The material experimentally demonstrates an exceptional effective absorption bandwidth of 5.1 GHz at an ultralow thickness of 1.0 mm, with an optimal reflection loss of −45.12 dB at a 1.9 mm. Furthermore, the formation of a protective Si─O─Si surface layer significantly enhances corrosion resistance, confirming practical durability. This study establishes an AI‐guided paradigm that successfully bridges electromagnetic theory with materials design, offering a robust and generalizable platform for the accelerated development of advanced microwave absorption materials.

## Graphical abstract

A neural network‐enabled permittivity engineering paradigm is introduced, transcending traditional trial‐and‐error design. By decoupling electromagnetic parameters and screening a high‐throughput feature space, an ultrathin (1.0 mm) magnetic absorber is inversely designed, experimentally achieving a superior and customizable 5.1 GHz bandwidth and robust corrosion resistance, thus bridging theory and AI‐guided material development.

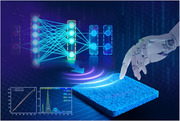

## 1 Introduction

Microwave absorbing materials occupy an indispensable role in high‐frequency communication systems, electromagnetic compatibility protection, and even modern defense technologies [1, 2]. With the ongoing trend toward miniaturization and integration in electronic devices, the development of broadband absorbers with ultrathin configurations has become increasingly important for applications in near‐field communication and Internet of Things (IoT) devices [3, 4]. This demand has become even more urgent, as modern radar detection systems continue to expand the operational frequency bands. Consequently, the creation of a new generation of ultrathin and broadband absorbing materials is crucial for advancing sophisticated electronic equipment and frontier stealth technologies [5, 6].

Magnetic absorbing materials are regarded as one of the most promising systems for achieving thin and lightweight absorption, owing to the additional magnetic loss mechanism [7]. Researchers have optimized the electromagnetic response properties through strategies such as microstructure regulation and multi‐component compositing [8, 9]. Among them, carbonyl iron powder has emerged as a core candidate for military absorbing coatings due to the high permeability and low coercivity [10, 11]. For instance, liquid metal‐coated carbonyl iron microspheres prepared via force‐induced techniques can achieve an optimal reflection loss of −65.94 dB at a thickness of 1.96 mm [12]. The adoption of laser ablation technology enables the combination of flaky carbonyl iron with tunable surface defects in carbon materials, leading to effective absorption across the S, X, and Ku bands at a matching thickness of 8.7 mm [13]. However, the intrinsic properties of magnetic materials impose limitations: permeability is constrained by the Snoek's limit, while the strong coupling between the frequency‐dependent complex permittivity and permeability makes it challenging to balance thin profiles and broadband absorption [14, 15]. Confronted with such complexity, the conventional development of absorbers heavily relies on trial‐and‐error methods or qualitative adjustments based on limited empirical knowledge. These approaches not only involve long development cycles and high costs but also lack universal theoretical guidance, making it difficult to systematically explore the full parameter space. As a result, material design often remains confined to local optimization, hindering substantial performance breakthroughs.

To overcome the limitations of conventional approaches, data‐driven research paradigms have emerged. In previous work by our team, the concept of a “permittivity genome” was proposed for non‐magnetic absorbing materials, establishing a quantitative mapping relationship between permittivity and absorption performance through numerical screening, which successfully guided material design [16]. However, extending this methodology to magnetic materials requires simultaneous consideration of frequency‐dependent permittivity and permeability, leading to an exponential expansion of the parameter space and imposing significant computational challenges in practice. Zhang et al. simplified the permeability as a fixed value while retaining only the dispersive permittivity — a simplified qualitative model that nonetheless sacrifices predictive accuracy for magnetic absorbers due to the neglect of magnetic dispersion [17]. Fortunately, the rapid advancement of AI technology offers new opportunities to address this challenge. In recent years, advanced algorithms such as support vector machines (SVM), random forests (RF), and neural networks (NN) have demonstrated considerable potential in materials science, owing to the powerful capabilities in feature recognition and nonlinear fitting [18, 19, 20]. Liu et al. employed a genetic algorithm to perform cross‐scale structural optimization of selectively cobalt‐coated carbon fibers, resulting in a lightweight and flexible microwave absorbing textile [21]. Zhong et al. utilized SVM to predict and optimize the integral values of the real and imaginary parts of the permeability in magnetic carbonyl iron/Fe_3_O_4_ composites, achieving model errors as low as 3.14% and −6.56%, respectively [22]. Yuan et al. applied the RF algorithm to achieve high‐precision prediction of the permittivity of graphene‐based flexible composites and aerogels, along with optimized multilayer structural design [23, 24]. Dong et al. integrated the whale optimization algorithm (WOA) with an NN model to co‐optimize dielectric polylactic acid/carbon fiber composite metamaterials, producing an eco‐friendly and degradable superstructure that exhibits both excellent absorption and mechanical performance [25]. As insightfully highlighted by Cao et al., machine learning is emerging as a transformative force in the field of absorbing materials [26, 27]. Therefore, developing a machine learning model that incorporates absorption mechanism and enables inverse design for magnetic absorbers represents a critical and urgent research gap that remains to be filled.

Herein, an NN‐based permittivity engineering strategy is developed to provide a customizable and highly efficient design platform for magnetic absorbing materials. An innovative design paradigm termed “permeability locking‐permittivity optimization” is introduced, which effectively decouples the interlinked electromagnetic parameters by adopting representative permeability as boundary conditions. Based on this approach, a high‐throughput permittivity feature space covering hundreds of millions of parameter combinations is constructed, followed by a dual‐task screening strategy that considers both ideal and effective absorption. Subsequently, a physics‐constrained NN‐based permittivity fitting model is established, capable of not only inversely predicting permittivity that satisfies target absorption performance with high accuracy, but also ensuring the plausibility of predictions through embedded physical rules. This data‐driven framework ultimately guides the inverse design and synthesis of flaky carbonyl iron/barium titanate composites. Experimental validation confirms that the obtained material achieves a remarkable absorption bandwidth of 5.1 GHz at an ultra‐thin thickness of 1.0 mm, along with excellent corrosion resistance. These results verify the effectiveness and practicality of the AI‐guided strategy from theoretical design to material realization, offering a highly versatile solution for the on‐demand design of advanced magnetic absorbers.

## 2 Results and Discussion

### 2.1 Construction of Permittivity Dataset

The transmission line model correlates the electromagnetic parameters of absorbing materials with their absorption performance. However, the multiparameter coupling renders the mapping exceedingly complex [28, 29]. Previously, our team established the “permittivity genome” for non‐magnetic materials via numerical screening and fitting, which successfully guided the design of such absorbing materials [16]. Nonetheless, for magnetic materials, the introduction of frequency‐dispersive relative complex permeability results in an exponential escalation of complexity [30].

**FIGURE 1:**
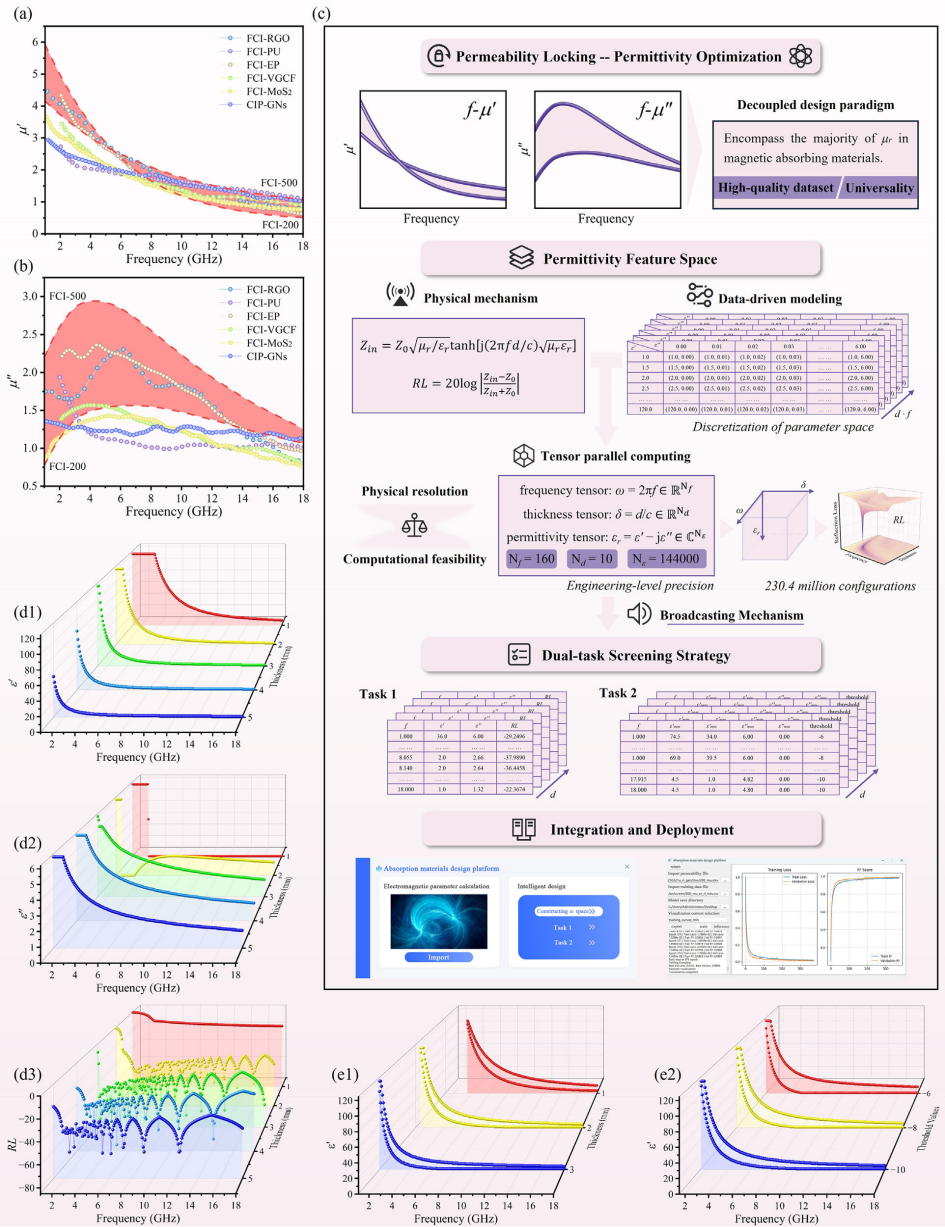
Reference ranges of (a) *µ'* and (b) *µ''* established by FCI‐200 and FCI‐500, with a comparison to the representative literature values [31, 32, 33, 34, 35, 36], (c) Schematic of the permeability locking‐permittivity optimization paradigm, tensor‐based permittivity space construction and dual‐task screening strategy, Representative screening results for (d1–d3) *ε'*, *ε''*, and the corresponding *RL* in Task 1, and (e1, e2) *ε'_max_* and *ε'_min_* in Task 2.

Herein, we introduce a decoupled design paradigm termed “permeability locking‐permittivity optimization” to overcome the challenges associated with the simultaneous optimization of conventional electromagnetic parameters. Taking ball‐milled carbonyl iron as an example, we constructed a high‐quality dataset for predicting the absorption performance of magnetic absorbing materials. Specifically, given that the permeability of current magnetic absorbing materials exhibits only minor variations with similar trends in 1–18 GHz, the permeability values of ball‐milled carbonyl iron measured at 200 rpm (FCI‐200) and 500 rpm (FCI‐500) were selected as reference boundary conditions (Figure 1a,b). These ranges effectively encompass the majority of *µ'* and a significant portion of *µ''* values encountered in magnetic absorbing materials [31, 32, 33, 34, 35, 36], thereby demonstrating a certain degree of universality. Notably, considering the demanding requirements for “ultrathin” and “broadband” absorption targets, the selected reference range for *µ''* is set slightly higher than the typical range of conventional carbonyl iron absorbers to enhance the magnetic loss effect.

Building on the aforementioned frequency‐dispersive relative complex permeability references, a high‐throughput screening framework based on tensor operations was developed to establish a permittivity feature space, effectively bridging the gap between the underlying physical mechanisms and data‐driven modeling requirements. Utilizing the transmission line model, four key variables were systematically integrated: frequency‐dispersive permeability (*µ_r_* = *µ'* − j*µ''*), permittivity (*ε_r_* = *ε'* − j*ε''*), material thickness (*d*), and operating frequency (*f*). The parameter space was discretized with engineering‐level precision:

(1) $$d\;\in \;\left[0.5,\;\;5.0\right],\;\Delta d=0.5\;mm$$

(2) $$\varepsilon^{\prime }\;\in \;\left[1.0,\;\;120.0\right],\;\Delta \varepsilon^{\prime }=0.5$$

(3) $$\varepsilon^{\prime \prime }\;\in \;\left[0.00,\;\;6.00\right],\;\Delta \varepsilon^{\prime \prime }=0.01$$

This strategy guarantees that each (*d*, *f*) configuration generates 1.44 × 10^6^ possible (*ε'*, *ε''*) combinations, achieving an optimal balance between computational feasibility and physical resolution. The absorption performance is quantified by calculating the reflection loss (*RL*), where *Z_0_* represents the free‐space impedance and *c* denotes the speed of light [8]:

(4) $$\;Z_{in}=Z_{0}\;\sqrt{\mu_{r}/\varepsilon_{r}}\tanh\left[j\left(2\pi fd/c\right)\sqrt{\mu_{r}\varepsilon_{r}}\right]$$

(5) $$RL=20\log\left|\frac{Z_{in}-Z_{0}}{Z_{in}+Z_{0}}\right|$$

The computational implementation leverages PyTorch's tensor processing paradigm to achieve hardware‐accelerated parallel computing, while 3D tensor operations replace traditional nested loops through broadcasting mechanisms:

(6) $$frequency\;tensor:\;\omega =2\pi f\;\in \;R^{N_{f}}$$

(7) $$thickness\;tensor:\;\delta =d/c\;\in \;R^{N_{d}}$$

(8) $$permittivity\;tensor:\;\varepsilon_{r}=\varepsilon^{\prime }\;-\;j\varepsilon^{\prime \prime }\;\in \;C^{N_{\varepsilon }}$$

The hyperbolic tangent argument was computed via Einstein summation (*ω*_{_ *_i_*_,_ *_j_*_,_ *_k_*_}_ *δ*_{_ *_j_*_,_ *_k_*_}_ $\sqrt{\mu_{r}\varepsilon_{r}}$), forming a complex tensor of dimensions [N*_f_* × N*_d_* × N*_ε_*]. Specifically, N*_f_* = 160, N*_d_* = 10, and N*_ε_* = 240 × 600, yielding a total parameter space of 230.4 million configurations.

Subsequently, a dual‐task screening strategy was applied to the generated *RL* results. The objective of Task 1 is to identify the optimal *RL* values. For each (*d*, *f*) pair, the (*ε'*, *ε''*) combination corresponding to the minimum *RL* is extracted along the *ε_r_* dimension, and the corresponding *RL* value is recorded. In practical engineering applications, achieving a broad effective absorption bandwidth (*EAB*) is often more desirable, as it significantly enhances the applicability of absorbing materials. Therefore, Task 2 focuses on identifying the effective *ε_r_* regions where *RL* falls below various threshold values (−10, −8, and −6), tailored to different application scenarios. For each (*d*, *f*, threshold) triplet, a Boolean mask is employed to extract the coordinates of the effective *ε_r_* region, followed by boundary retrieval to determine the upper and lower limits of *ε'* and *ε''*. This process establishes a data‐driven foundation for the broadband design of absorbing materials. As illustrated in Figure 1c, a visual overview is provided for the construction of the permittivity feature space and the execution of the dual‐task screening strategy, along with the dataset format for each task. Examples from the dual‐task screening strategy are presented in Figure 1d,e, with the complete results available in Figure S1. The entire computation was performed on an NVIDIA RTX 4070 Ti GPU, taking only 10.08 s — achieving a speedup of several thousand times compared to traditional CPU‐based nested loop implementations.

### 2.2 NN‐Based Permittivity Fitting Model

In contrast to the permittivity fitting of non‐magnetic absorbing materials, the permittivity dataset obtained through the dual‐task screening strategy (Figure 1d,e; Figure S1), which originates from the permeability of FCI‐200 and FCI‐500, is observed to exhibit intricate nonlinear behavior that poses significant challenges for conventional function fitting approaches. The inherent complexity of these trend profiles renders mathematical parameterization impractical, as the derived functional forms typically lack conciseness and demonstrate limited utility in guiding materials design. As a robust strategy for addressing nonlinear regression problems in deep learning, neural networks can offer a transformative solution for correlating complex electromagnetic parameters through the inherent capabilities for autonomous multi‐level feature extraction and data‐driven pattern recognition. This artificial intelligence paradigm has been strategically implemented to establish an NN‐based permittivity fitting model, specifically engineered for the inverse intelligent design of magnetic absorbing materials with carbonyl iron as the representative system.

**FIGURE 2:**
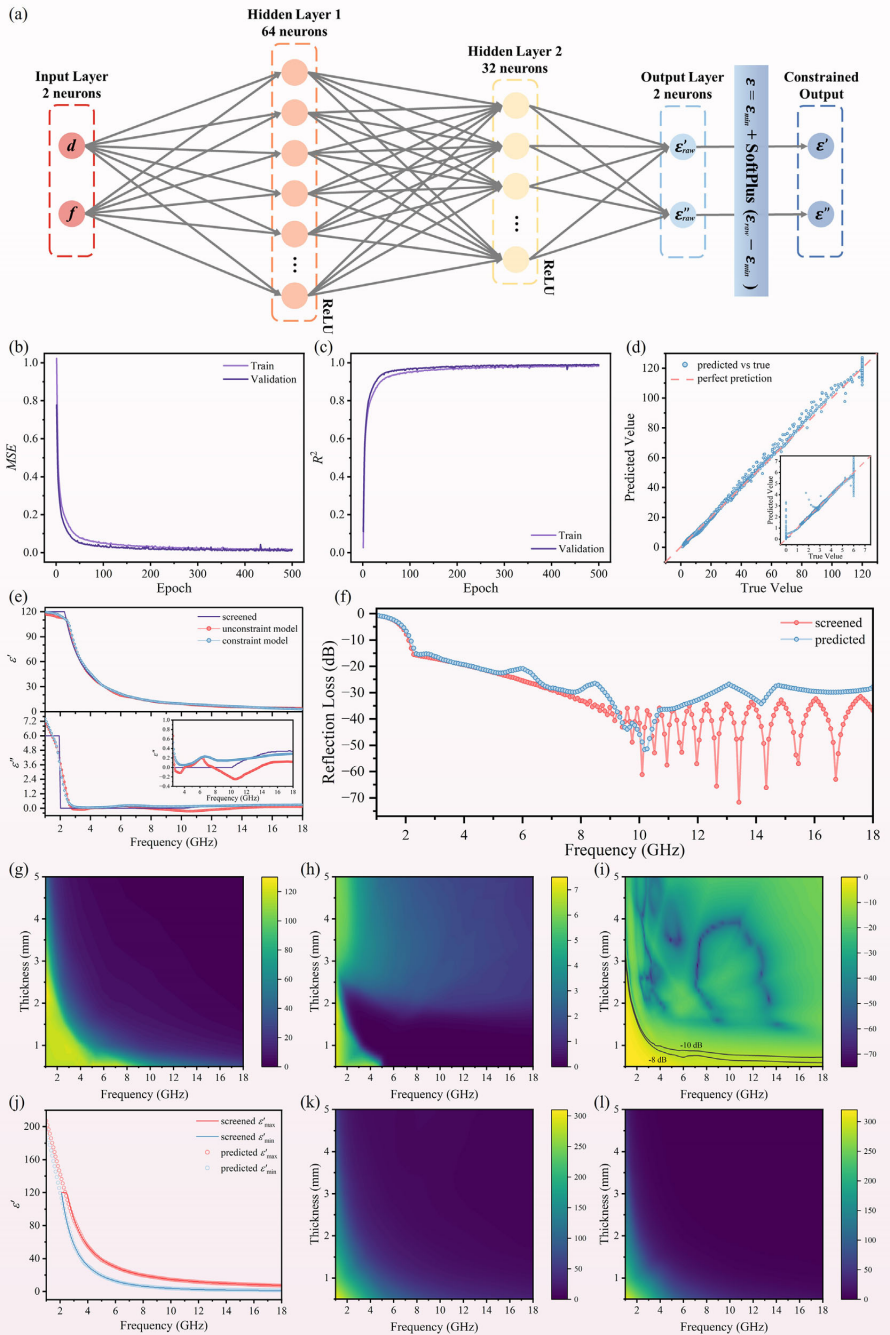
(a) Architecture of the NN‐based permittivity fitting model, (b) *MSE* and (c) *R^2^* curves on the FCI‐200 (Task 1), (d) Scatter plots of predicted values vs. true values for *ε'* and *ε''* (inset), (e) Comparison of the predicted *ε'* and *ε''* from the constrained and unconstrained models against the screened results for FCI‐200 at 1.5 mm, (f) Comparison of the corresponding *RL* between the model inference and screened data, Model‐inferred solution sets for ideal absorption: heatmaps of feasible (g) *ε'*, (h) *ε''*, and (i) the corresponding *RL*, (j) Comparison of the predicted *ε'_max_* and *ε'_min_* from the model against the screened results for FCI‐200 at 1.5 mm, Model‐inferred solution sets for effective absorption (RL < −10 dB): heatmaps of feasible (k) *ε'_max_* and (l) *ε'_min_*.

Based on the dataset from Task 1, the prediction of permittivity corresponding to optimal *RL* values has been first conducted. A multilayer network with two hidden layers was constructed, featuring 2 input neurons (*d* and *f*) and 2 output neurons (predicted *ε'* and *ε''*). The network architecture adopted a fully connected configuration with 64 neurons and 32 neurons in successive hidden layers, employing ReLU activation functions to enhance nonlinear mapping capabilities (Figure 2a). The dataset was partitioned into training, validation, and test subsets at a ratio of 7:2:1. To ensure numerical stability and accelerate model convergence, rigorous standard normalization was implemented on both input features and output parameters through z‐score transformation:

(9) $$X_{scaled}=\frac{X-\mu_{X}}{\sigma_{X}}\;$$

(10) $$y_{scaled}=\frac{y-\mu_{y}}{\sigma_{y}}\;$$

where *X* denotes the input features encompassing *d* and *f*, and *y* corresponds to the output parameters *ε'* and *ε''*. Complementing the architectural design, the hyperparameters were optimized through systematic parameter space exploration. The training protocol was configured with the following critical parameters: batch size [64], initial learning rate (0.001), and training epochs (500), employing the Adam optimizer for its adaptive learning rate advantages in complex parameter space navigation. To rigorously quantify the model accuracy, a mean squared error (*MSE*) loss function was mathematically formulated as [37]:

(11)

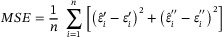

where *n* represents the batch size, *y_i_* and ${\hat{y}}_{i}$ correspond to the normalized truth label and predicted value for sample *i*, respectively. Simultaneously, the early stopping mechanism was configured with a patience of 50 epochs, automatically halting training when no improvement in validation loss was observed for consecutive epochs to prevent overfitting and conserve computational resources. To further improve the model's generalization capability and address the challenge of physical plausibility when extrapolating beyond the training data distribution, a fundamental physical constraint was incorporated into the network architecture through a SoftPlus activation transformation (Figure 2a). SoftPlus function as a smooth approximation of the ReLU function that ensures stable gradient backpropagation for fitting complex non‐linear curves. Meanwhile, unlike physics‐informed inequality loss terms that merely suppress negative values, the approach utilizing SoftPlus enables dynamic adaptation of output boundaries governed by the statistical properties of the training dataset, while rigorously confining the predicted permittivity values within physically meaningful ranges, thereby eliminating the need for manual hyperparameter tuning. This mathematical non‐negativity constraint ensures a robust model architecture that intrinsically integrates physical principles with a data‐driven paradigm.

As depicted in Figure 2b,c, using the dataset from Task 1 under the 200 rpm condition, the model demonstrates exceptional performance. Specifically, both training and validation sets exhibit a consistently decreasing trend in *MSE* with increasing epoch numbers, ultimately converging to a low value of 0.0140. Meanwhile, the validation set achieves an impressive *R* ^2^ score of 0.9886. Furthermore, the comparative scatter plots between predicted and truth values (Figure 2d; Figure S2) reveal robust predictive capability for both *ε'* and *ε''*. However, due to the restricted range of *ε''* intentionally defined during parameter space construction to reflect practical material properties, the model exhibits minor deviations in predictions near the boundaries of the *ε''* domain. This constrained *ε''* selection introduces a slight edge effect while ensuring physical relevance.

As further evidenced by Figure 2e, the model demonstrates enhanced physical plausibility through the SoftPlus constraint, resulting in permittivity predictions that align perfectly with true values. Although the physics‐agnostic model also exhibits comparable predictive accuracy, the generated permittivity results, especially the *ε''*, display negative values, which violate fundamental physical laws. Crucially, the precise prediction of permittivity is not the ultimate goal, and the electromagnetic theoretical calculations are carried out subsequently. Converting the predicted *ε'* and *ε''* into *RL* results through integrated transmission line theory, and judging whether the absorption performance achieves the optimal effect, is important evidence to demonstrate the effectiveness of the model. Correspondingly, the calculated *RL* based on the predicted permittivity is shown in Figure 2f (complete results of permittivity and *RL* in Figure S3), which maintains excellent consistency with the screening results in low‐frequency regimes, while achieving ideal absorption (*RL* < −20 dB) across high‐frequency domains. To enhance practical utility in material design, the thickness resolution was refined from 0.5 to 0.1 mm, enabling full‐parameter‐space inference through the NN‐based permittivity fitting model. This advancement generates comprehensive distributions of *ε'*, *ε''*, and validated *RL* mapping across refined multidimensional spaces (Figure 2g–i), which visually decouple the complex interplays among magnetic carbonyl iron's permittivity values, geometric thicknesses, operational frequencies, and microwave absorption characteristics. Such high‐dimensional visual analytics establish a quantitative framework for identifying optimal absorption zones. Notably, the model forms closed‐loop verification based on physical information compared to conventional machine learning methods, ensuring both designability and high efficiency.

Similarly, the fitting model architecture for the Task 2 dataset maintains structural consistency with Task 1. The evaluation revealed that for any of the three labels corresponding to the effective absorption regions in Task 2, the *ε''* values were almost entirely satisfied within the predetermined range (Figure S1), where the upper and lower boundaries of the effective region for *ε''* were identified as 6 and 0, respectively. Consequently, only the boundaries of *ε'* were fitted for prediction. The dataset of Task 2 is extracted according to the (−10, −8, and −6) label, and the above NN‐based permittivity fitting model is employed three times respectively to generate distinct *ε'* solution spaces for each absorption threshold. While retaining the 2 output neurons structure from Task 1, the model now predicts the upper and lower bounds of *ε'* in the effective region under various effective absorption conditions, rather than predicting the *ε'* and *ε''* values associated with ideal absorption scenarios. Notably, to prevent the illogical scenario where upper bounds might dip below lower bounds, the datasets for both predictors are jointly normalized rather than undergoing separate normalization alone in Task 1. The model demonstrates outstanding performance and robust generalizability, achieving a remarkably *R* ^2^ score of 0.9983 on the 200 rpm validation set for *RL* < −10 dB targets. As shown in Figure 2j, the predicted *ε'* boundaries exhibit exceptional congruence with screening results (complete dataset in Figure S4). Also, the parameter space was refined and reconstructed by inference, and the mapping results of *ε'* upper and lower boundaries were obtained (Figure 2k,l), establishing a theoretical foundation for the prediction of various target effective regions and the customized design of absorption performance. The model exhibited consistent performance on the dataset under the 500 rpm condition, as detailed in Figures S5 and S6.

In summary, this dual‐task screening strategy, synergized with the NN‐based permittivity fitting model, successfully maps both ideal and effective absorbing performance spaces, providing a more convenient and intelligent new design strategy for absorbing materials.

### 2.3 Design Validation of FCI‐Based Absorber

As revealed by the NN‐based permittivity fitting model, the permittivity of FCI‐500 at low thickness is significantly higher than the solution set corresponding to the optimal absorption performance. This result underscores the versatility of the proposed “permeability locking‐permittivity optimization” strategy: by inputting the permeability boundaries of a specific magnetic filler, the framework can identify the matching permittivity space, thereby guiding the rational selection of second‐phase materials for modification. Specifically for the FCI system, the model explicitly indicates that to resolve the impedance mismatch caused by high conductivity, it is essential to reduce *ε''* as much as possible, while maintaining *ε'* at a relatively high level. Guided by this data‐driven prediction, BaTiO_3_, a common ferroelectric ceramic material [38], was identified as the ideal candidate. It inherently features a high *ε'* and a low *ε''*, which aligns perfectly with the optimal parameters targeted by the neural network. However, a simple co‐ball‐milled system remains a dispersed phase, which leads to inaccuracies in electromagnetic parameter measurements and limited effectiveness in reducing *ε''*. Therefore, KH560 and DMAOP were employed to bridge FCI and BaTiO_3_. This approach also facilitates the formation of a thin Si─O─Si layer on the surface, which not only helps tailor the permittivity but also enhances anti‐corrosion performance [39].

**FIGURE 3:**
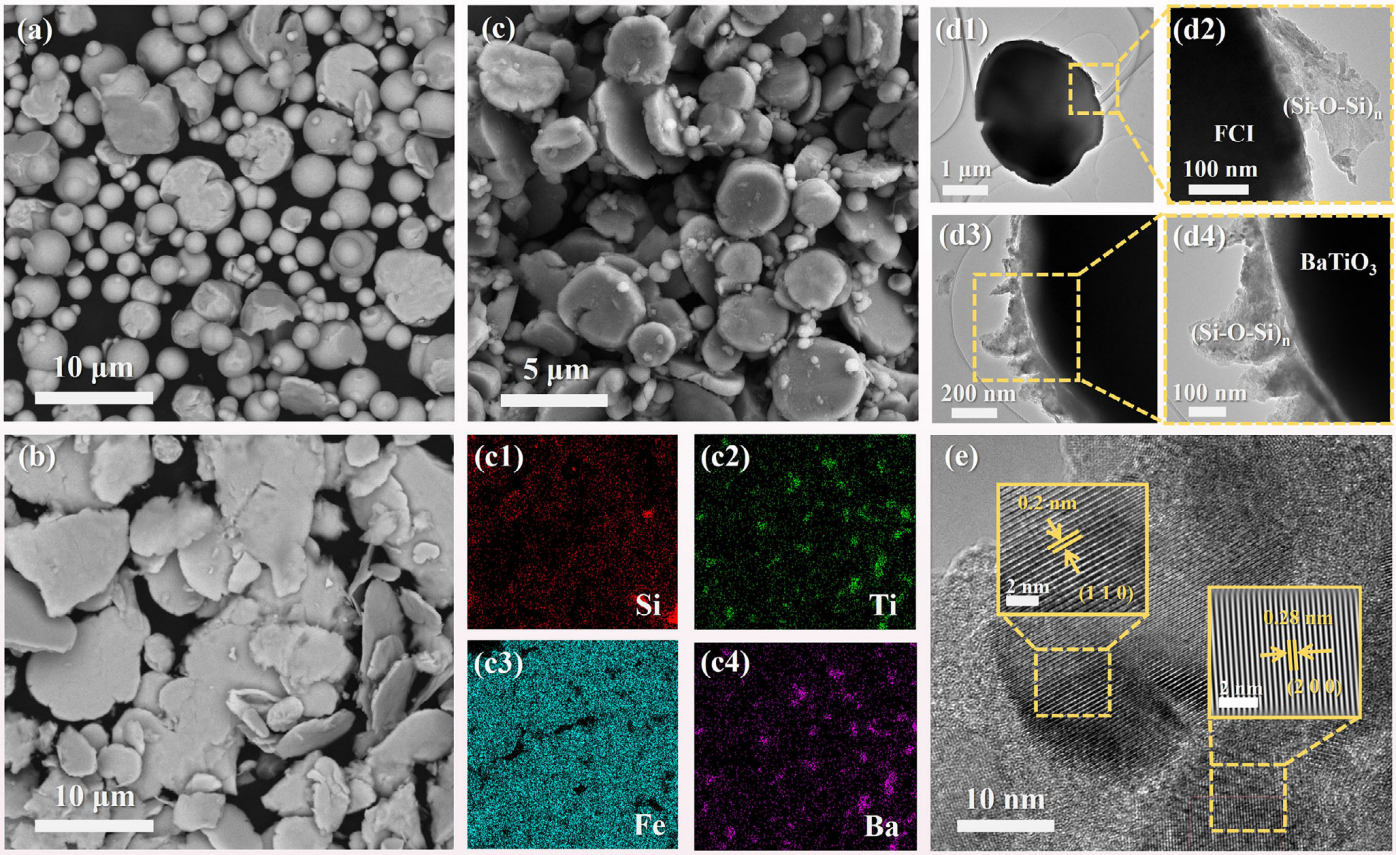
SEM images of (a) FCI‐200, (b) FCI‐500, (c) FCI‐BT and corresponding EDS mappings of (c1) Si, (c2) Ti, (c3) Fe, and (c4) Ba elements in the respective region, (d1–d4) TEM and (e) high‐resolution TEM images of FCI‐BT.

As shown in Figure 3a, when the ball milling rotation speed was set at 200 rpm, the CIP particles largely retained their spherical morphology, with some flakes still exhibiting considerable thickness, which is unfavorable for mitigating the skin effect. When the rotation speed was increased to 500 rpm (Figure 3b), the spherical particles transformed into thin flakes. This flattened morphology offers a larger aspect ratio and specific surface area, facilitating the formation of interconnected conductive networks among the flaky carbonyl iron (FCI) particles, thereby enhancing microwave absorption [40]. The introduction of BaTiO_3_ effectively prevented over‐flaking of the CIP particles caused by excessive energy input during high‐speed ball milling and reduced direct contact between FCI flakes (Figure 3c). From the perspective of average particle size, the mean sizes of FCI‐200 and FCI‐500 were 3.47 and 6.95 µm, respectively, while the incorporation of BaTiO_3_ resulted in an intermediate FCI size of 4.58 µm, indicating a moderating effect. Furthermore, EDS analysis (Figure 3c1–c4) revealed that after 8 h of co‐ball milling, BaTiO_3_ was uniformly distributed among the FCI particles without agglomeration. This confirms the successful bridging between FCI and BaTiO_3_ via DMAOP, as well as the formation of a thin SiO_2_ coating on both material surfaces. This microstructure provides a foundation for tuning the electromagnetic parameters and optimizing wave‐absorbing performance, while also improving corrosion resistance. Correspondingly, the TEM result (Figure 3d1–d4) further confirmed the presence of a thin SiO_2_ layer on both FCI and BaTiO_3_, consistent with the EDS findings. As shown in Figure 3e, the measured interplanar spacings in the enlarged region were 0.2 and 0.28 nm, corresponding to the (110) and (200) crystal planes of carbonyl iron, respectively. This indicates that the ball milling process primarily alters the morphology of CIP without changing its crystal structure, thus preserving its intrinsic physical properties [41].

**FIGURE 4:**
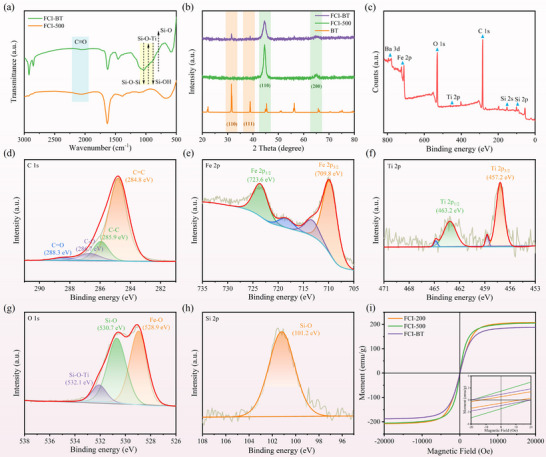
(a) FTIR spectra and (b) XRD patterns of different samples, (c) XPS spectrum of FCI‐BT, (d–h) High‐resolution XPS spectra of FCI‐BT in the C 1s, Fe 2p, Ti 2p, O 1s, and Si 2p regions, (i) Magnetic hysteresis loops of different samples.

The FCI‐BT composite powder was characterized by FTIR spectroscopy. As shown in Figure 4a, the C≡O stretching vibration (ν(CO)) of pure Fe(CO)_5_ appears as a broad band near 2049 cm^−1^. After compositing with BaTiO_3_, this peak is significantly attenuated, indicating successful replacement of the C≡O ligands by DMAOP during the reaction. The peak at 1086 cm^−1^ is attributed to the Si─O─Si stretching vibration. In the composite system, this peak broadens and shifts to a lower wavenumber, suggesting hydrolysis of methoxy groups to form Si─OH, followed by condensation with the BaTiO_3_ surface to form Si─O─Ti bonds, as evidenced by the peak at 958 cm^−1^. The broadened Si─O─Si peak indicates the formation of a siloxane network [42, 43], while the newly emerged Si─O─Ti peak confirms the successful chemical modification of the BaTiO_3_ interface by KH560 and DMAOP.

XRD analysis (Figure 4b) reveals characteristic diffraction peaks corresponding to the perovskite phase of BaTiO_3_. The sharp peaks at 31.5° and 38.9° are indexed to the (110) and (111) crystal planes, respectively, confirming its tetragonal structure [44]. For FCI‐500, the diffraction peaks at 44.6° and 65.1° correspond to the (110) and (200) planes of body‐centered cubic (bcc) carbonyl iron, reaffirming the preservation of the crystal structure after high‐speed ball milling [45]. The XRD pattern of the FCI‐BT composite clearly shows a combination of characteristic peaks from both BaTiO_3_ and carbonyl iron, providing strong evidence for the coexistence of the two phases. No additional diffraction peaks or noticeable peak shifts are observed, though the two characteristic peaks of carbonyl iron exhibit slight broadening. It suggests that the KH560 modification and DMAOP incorporation did not induce phase transformation, but may have introduced localized dislocations in the carbonyl iron after co‐ball milling. In summary, the modified BaTiO_3_ and carbonyl iron have been successfully integrated while maintaining their structural integrity, laying a solid foundation for the tailored design of electromagnetic performance.

The XPS spectrum of the FCI‐BT composite (Figure 4c) exhibits two distinct peaks at 457.2 and 462.5 eV, confirming the presence of Ti originating from BaTiO_3_. In the high‐resolution C 1s spectrum (Figure 4d), the peaks located at 284.8, 285.9, 286.7, and 288.3 eV are assigned to four functional groups in FCI‐BT: C═C, C─C, C─O, and C═O, respectively [46]. The high‐resolution Fe 2p spectrum (Figure 4e) displays binding energies at 723.6 and 709.8 eV for the Fe 2p_1/2_ and Fe 2p_3/2_ orbitals, respectively, which originate from the Fe─C bonds in CIP [47]. The Ti 2p spectrum (Figure 4f) reveals two peaks with different binding energies: the peak at 457.2 eV is attributed to the Ti═O bond, while the peak at 463.2 eV corresponds to the Ti─O bond [48]. The O 1s high‐resolution spectrum (Figure 4g) further indicates the successful bridging of FCI and BaTiO_3_ via silicon. Moreover, the Si 2p spectrum (Figure 4h) shows a characteristic peak at 101.2 eV, confirming the presence of Si─O bonds [49]. Collectively, the XPS results provide additional evidence for the formation of a Si─O─Si network and the successful bridging of BaTiO_3_, which is consistent with the FTIR results.

Subsequently, an in‐depth analysis of the magnetic properties of the material was carried out. Theoretically, the initial permeability (*µ_i_*) of the material is influenced by intrinsic parameters such as saturation magnetization (*Ms*), coercivity (*Hc*), magnetostriction coefficient (*λ*), and elastic strain parameter (*ξ*). The detailed relationship is given by [50]:

(12) $$\mu_{i}=\frac{M_{s}^{2}}{akH_{c}M_{s}+b\lambda \xi }\;$$

where *a* and *b* are geometric factors of the material, and *k* is the domain wall pinning coefficient, which reflects the pinning strength of internal defects on magnetic domain wall motion. In addition, *µ_i_* is closely related to the magnetization precession of internal magnetic dipoles under an external magnetic field, as described by the Snoek's limit [51]:

(13) $$\left(\mu_{i}-1\right)\;f_{r}=\frac{2}{3\pi }\;\gamma \times 4\pi M_{s}$$

here, *γ* represents the gyromagnetic ratio, and *f_r_* denotes the natural resonance frequency. Owing to the Snoek*’*s limit, *µ_i_* drops sharply when the operating frequency exceeds the gigahertz range, while *Ms* also serves as a critical factor influencing *µ_i_*. The hysteresis loops of FCI‐200, FCI‐500, and FCI‐BT are presented in Figure 4i, illustrating the magnetic performance under different external magnetic fields. FCI‐200 exhibits typical ferromagnetic behavior with relatively low *Hc* and high *Ms*. In contrast, FCI‐500 shows increased *Hc* and a slight reduction in *Ms*, indicating that the ball milling process introduced internal strain and dislocations, resulting in a more disordered crystal structure along with enhanced magnetic anisotropy and domain wall pinning effects. Notably, FCI‐BT displays a distinct hysteresis loop characterized by increased *Hc* and a moderate decrease in *Ms*. This behavior can be attributed to the interfacial interaction between carbonyl iron and modified BaTiO_3_, as well as the surface modification by DMAOP, which collectively introduce additional magnetic domain pinning sites and reduce the overall magnetic moment. These findings demonstrate that both ball milling and surface modification allow fine‐tuning of the magnetic property without altering the crystal structure, thereby offering a feasible strategy for the customized design of microwave absorption performance [52].

**FIGURE 5:**
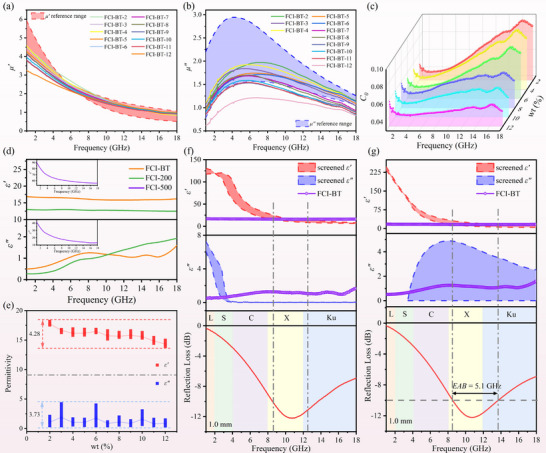
(a,b) Frequency dependence of *µ'* and *µ''* for all samples and the reference ranges, (c) Comparison of *C_0_* for different samples, (d) Frequency dependence of *ε'* and *ε''* for different samples, (e) Tunable ranges of *ε'* and *ε''* for FCI/BaTiO_3_ composites with different mass ratios, (f,g) Validation of the model for Task 1 and Task 2 using FCI‐BT at 1.0 mm.

Subsequently, customized design validation was performed for magnetic FCI‐based absorbers using the NN‐based permittivity fitting model. Briefly, *µ′* and *µ″* reflect the ability to store and dissipate magnetic energy, respectively [53]. As shown in Figure 5a,b, the *µ′* values of all samples decrease with increasing frequency, exhibiting notable dispersion behavior. The introduction of BaTiO_3_ has a minor influence on *µ′*, and all FCI samples maintain good magnetic energy storage capability. Meanwhile, increasing the BaTiO_3_ content gradually reduces *µ″*. Overall, the *µ′* and *µ″* values of most samples fall within the reference region bounded by FCI‐200 and FCI‐500, strongly confirming the effectiveness of the selected permeability reference dataset.

Notably, the *µ″* curves of all samples exhibit slight fluctuations in the frequency ranges of 4–9 and 15–17 GHz, suggesting the coexistence of multiple magnetic loss mechanisms such as exchange resonance, eddy current effect, and natural resonance. The magnetic loss mechanism can be evaluated using the *C_0_* value, expressed as [51]:

(14) $$C_{0}=\mu^{\prime \prime }\;{\left(\mu^{\prime }\right)}^{-2}f^{-1}$$

It is generally accepted that when eddy current effects dominate, *C_0_* remains nearly constant over a certain frequency range, whereas significant variations in *C_0_* indicate the prevalence of exchange resonance [54]. As illustrated in Figure 5c, the *C_0_* values of different samples vary considerably with frequency in the 14–18 GHz range, which is primarily attributed to exchange resonance. In contrast, in the low‐frequency region of 2–5 GHz, *C_0_* shows minimal variation, suggesting that magnetic loss in this range arises from a combination of natural resonance and exchange resonance.

Following the “permeability locking‐permittivity optimization” strategy, it can be observed from Figure 5d that the permittivity can be precisely tuned by adjusting the mass fraction of BaTiO_3_. As the BaTiO_3_ content increases, the *ε′* value gradually decreases, with a total tunable range of 4.28 (from 18.17 to 13.89). Similarly, the *ε″* value also exhibits a tunable range of 3.73. Such flexible and controllable permittivity adjustment provides significant convenience for the customized design of absorbing performance.

The NN‐based permittivity fitting model was validated using a typical FCI‐BT sample (FCI‐BT‐5). As shown in Figure 5e, compared to FCI‐200, both *ε′* and *ε″* of FCI‐500 increase significantly, which is mainly attributed to the excessive flaking of CIP during ball milling, leading to the formation of more conductive pathways and enhanced interfacial polarization. However, such excessively high permittivity results in an unfavorable impedance mismatch, substantially compromising the absorbing potential. Introducing BaTiO_3_ as a second component at 500 rpm effectively mitigates the over‐flaking process during ball milling, enhancing interfacial polarization while avoiding the impedance mismatch caused by high conductive loss. It is evident that compared to FCI‐500, both *ε′* and *ε″* of FCI‐BT are significantly reduced, with *ε″* approaching zero, which aligns well with the theoretical values calculated by the NN‐based permittivity fitting model.

For FCI‐BT, the validation of ideal absorption (Task 1) was first conducted at a thin thickness of 1.0 mm. The feasible optimal *RL* region corresponds to the intersection between the measured *ε′* and the theoretically predicted region, as well as that between *ε''* and the theoretical domain. Given that the theoretical *ε''* approaches zero under thin‐thickness conditions — a criterion difficult to strictly satisfy in practice — a sufficiently low *ε''* value was considered acceptable. As shown in Figure 5f, the *ε′* values of FCI‐BT fall within the theoretical region over 8.65–12.56 GHz, and the *RL* curve confirms favorable absorption performance precisely in this frequency range. Subsequently, for Task 2 targeting effective absorption, a threshold of −10 dB was adopted for validation. As illustrated in Figure 5g, the *ε''* curve lies entirely between the upper and lower theoretical bounds across 4–18 GHz, while *ε′* overlaps with the model‐predicted region from 8.57 to 13.67 GHz. This frequency range aligns perfectly with the observed RL < −10 dB band. These results strongly validate the accuracy of the NN‐based permittivity engineering strategy. Moreover, the tailored magnetic absorber achieves an *EAB* of 5.1 GHz at only 1.0 mm, demonstrating significant practical potential.

**FIGURE 6:**
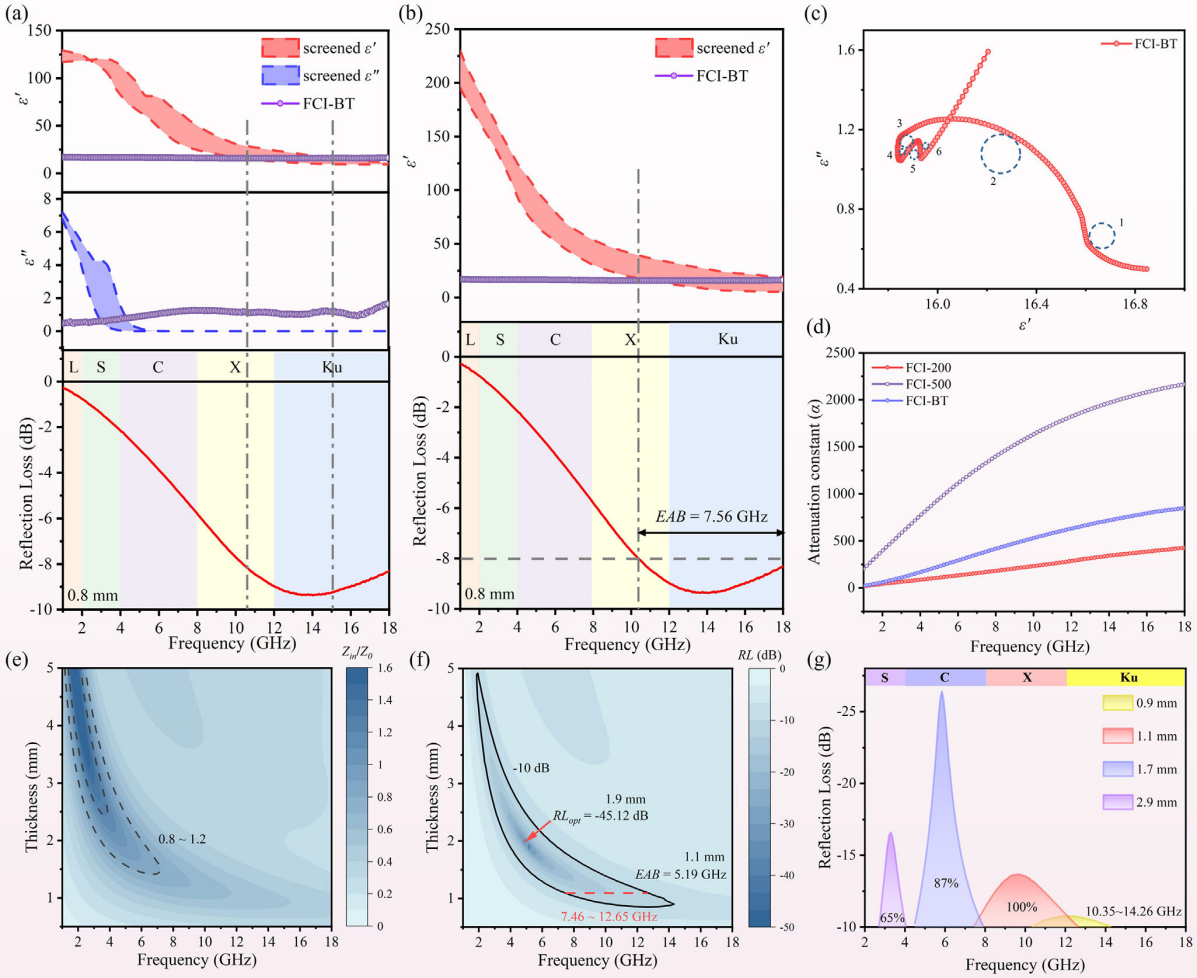
(a,b) Generalization validation of the model for Task 1 and Task 2 using FCI‐BT at 0.8 mm, (c) Cole‐Cole plot of FCI‐BT, (d) Comparison of *α* for different samples, (e) 2D mapping of |*Z_in_*/*Z_0_* | for FCI‐BT with different thicknesses, (f) 2D *RL* mapping of FCI‐BT with different thicknesses, (g) Customizable absorption performance of FCI‐BT with different thicknesses.

To further investigate the generalization capability of the NN‐based permittivity fitting model, the absorption performance of FCI‐BT was validated at a reduced thickness of 0.8 mm. As shown in Figure 6a,b, the overlapping permittivity regions align well with the dual‐task objectives. Here, Task 2 adopted a threshold of −8 dB for validation. Under such an extreme thickness condition of 0.8 mm, FCI‐BT still achieves an *EAB* (*RL* < −8 dB) of 7.56 GHz in the 10.44–18 GHz range, demonstrating both remarkable broadband absorbing performance and the generalizability of the NN‐based permittivity engineering method.

Furthermore, the absorbing performance was analyzed from a physical perspective to provide deeper insights into the model predictions. Cole‐Cole semicircles (Figure 6c; Figure S7) offer a visual representation of polarization responses [55]. Compared to pure FCI, the FCI‐BT exhibits more pronounced polarization processes and stronger microwave response, which can be attributed to the increased defect sites induced by localized dislocations during ball milling. These defects promote dipole formation and polarization, while the incorporation of BaTiO_3_ introduces additional heterogeneous interfaces, enhancing interfacial polarization.

Achieving optimal microwave absorption requires a balance between impedance matching and loss performance. The attenuation constant (*α*) serves as an indicator of loss capability, expressed as [56, 57]:

(15) $$\alpha =\frac{\sqrt{2}\pi f}{c}\sqrt{\mu^{\prime \prime }\varepsilon^{\prime \prime }-\mu^{\prime }\varepsilon^{\prime }+\sqrt{{\left(\mu^{\prime \prime }\varepsilon^{\prime \prime }-\mu^{\prime }\varepsilon^{\prime }\right)}^{2}+{\left(\varepsilon^{\prime }\mu^{\prime \prime }+\varepsilon^{\prime \prime }\mu^{\prime }\right)}^{2}}}$$

And the *Z_in_*/*Z_0_* is used to evaluate impedance matching effectiveness [58]. As illustrated in Figure 6d, FCI‐BT exhibits a higher *α* value than FCI‐200, indicating superior electromagnetic wave dissipation. In contrast, although FCI‐500 shows a higher *α*, its severe impedance mismatch (Figure S8) prevents electromagnetic waves from entering the material interior, leading to poor absorption performance (Figure S9). As shown in Figure 6e, FCI‐BT also demonstrates excellent impedance matching, with a larger proportion of *Z_in_*/*Z_0_* falling within the optimal range of 0.8–1.2, particularly extending into the low‐frequency region, which indicates favorable absorption behavior.

In terms of absorption performance (Figure 6f), FCI‐BT achieves a widest *EAB* of 5.19 GHz at a thickness of 1.1 mm. Even at 1.0 mm, it maintains an *EAB* of 5.1 GHz. Moreover, at 1.9 mm, an optimal *RL* of −45.12 dB is observed at 5.16 GHz. Impressively, by simply varying the thickness of FCI‐BT, customizable absorption across different frequency bands can be flexibly achieved (Figure 6g). For instance, full X‐band coverage is realized at only 1.1 mm. Additionally, the FCI‐BT absorber can cover 87% of the C‐band at 1.7 mm and 65% of the S‐band at 2.9 mm, effectively addressing the challenge of low‐frequency absorption at small thicknesses. Compared to pure FCI, this absorber enables broader frequency coverage at significantly reduced thicknesses (Figure S9), highlighting the substantial practical value in facilitating the development of thin, wideband absorbing coatings.

**FIGURE 7:**
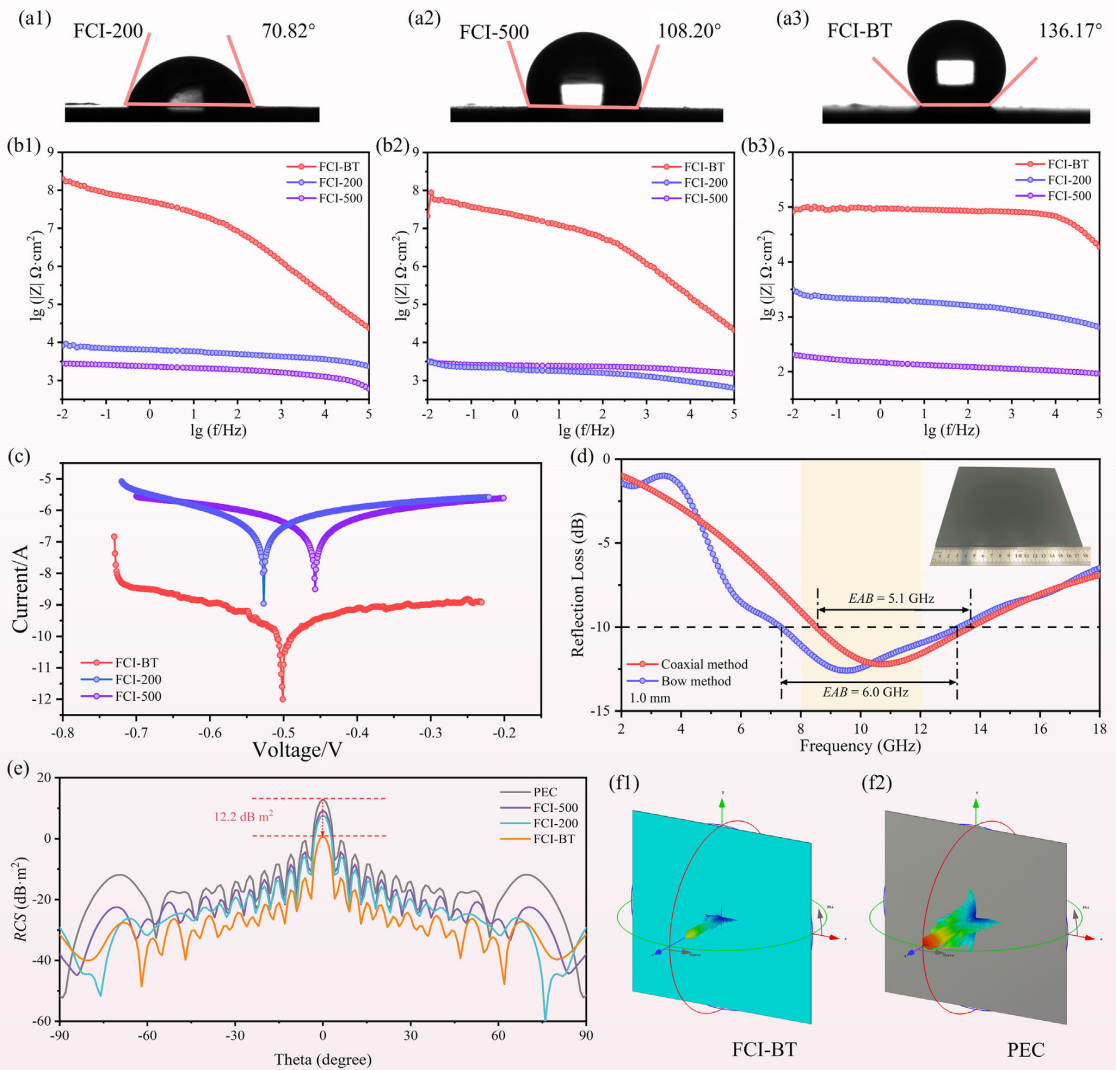
(a1–a3) Contact angles of FCI‐200, FCI‐500, and FCI‐BT, (b1–b3) Bode impedance modulus plots of different samples after salt spray testing for 3 days, 5 days, and 7 days, (c) Tafel polarization curves after 7 days of salt spray exposure, (d) 2D *RL* of FCI‐BI measured by coaxial and bow methods, and the inset is an optical photograph of the bow method specimen, (e) 2D *RCS* simulation results for different samples, (f1, f2) 3D far‐field *RCS* simulation patterns for FCI‐BT and PEC.

In practical applications, absorbing fillers are typically incorporated into an epoxy resin matrix to form functional coatings for microwave absorption. Under typical service conditions, a great corrosion resistance of the coating is essential to ensure long‐term and stable absorbing performance [59]. Generally, hydrophobicity plays a positive role in improving the corrosion resistance of coatings [60]. Herein, the wettability of the samples was characterized by measuring the water contact angle at room temperature. As shown in Figure 7a1, the contact angle of FCI‐200 is only 70.82°, indicating hydrophilic behavior. In contrast, FCI‐500, obtained under high‐speed ball milling, exhibits a flake‐like morphology with a high aspect ratio. The resulting nano‐micro structure contributes to a hydrophobic character, yielding a contact angle of 108.20° (Figure 7a2). For FCI‐BT, the presence of a thin Si─O─Si surface layer further enhances hydrophobicity, with a contact angle reaching 136.17° (Figure 7a3), demonstrating remarkable corrosion resistance potential. The corrosion resistance of the coatings was further investigated using electrochemical measurements. As shown in Figure 7b1–b3, the impedance modulus of Q235 steel coated with different coatings after 3 days, 5 days, and 7 days of salt spray testing. In general, the impedance modulus in the low‐frequency region is widely used to evaluate the corrosion protection performance of coatings, where a higher value indicates better performance [61]. Compared to the pure FCI coatings, the FCI‐BT coating exhibits a significantly increased impedance modulus, retaining a value of 10^5^ Ω·cm^2^ in the low‐frequency region even after 7 days. Furthermore, in the Bode phase angle plots (Figure S10), the FCI‐BT coating shows a higher phase angle in the high‐frequency region, suggesting a greater number of dielectric diffusion paths and improved barrier effect against corrosive species [62]. The Tafel polarization curves of the coatings after 7 days of salt spray exposure are presented in Figure 7c. The FCI‐BT coating displays a notably lower corrosion current density compared to the pure FCI coatings, indicating significantly enhanced corrosion resistance. This improvement can be mainly attributed to the barrier effect of the Si─O─Si layer, which effectively inhibits the interaction between corrosive media and the FCI fillers. In addition, the adhesion of BaTiO_3_ to the metal surface further hinders the access of corrosive ions to the substrate. These combined effects endow the FCI‐BT coating with excellent corrosion resistance, ensuring its reliability in long‐term service.

To further demonstrate the practical performance of the FCI‐BT coating, a metal substrate measuring 180 mm × 180 mm was uniformly spray‐coated with a 1 mm‐thick FCI‐BT layer and evaluated using the bow method, as shown in Figure 7d. This result aligns with the finding obtained from the coaxial method, confirming that the coating achieves full absorption across the X‐band at an ultra‐low thickness. Furthermore, radar cross section (*RCS*) simulations of the coating were performed using CST Microwave Studio to characterize its far‐field electromagnetic scattering behavior. The coating panel was modeled with dimensions of 180 mm × 180 mm, excited at a frequency of 10.8 GHz, with a 1 mm‐thick absorbing layer backed by a perfect electric conductor (PEC). The *RCS* results quantify the intensity of radar echoes reflected from a target, which is a key metric for radar detectability—a higher *RCS* corresponds to greater radar visibility [63, 64]. As depicted in Figure 7e, the FCI‐BT coating exhibits a notable *RCS* reduction of 12.2 dB·m^2^ at theta = 0° relative to the uncoated target, outperforming the other coatings. This significant performance is further visualized in the 3D far‐field simulation results in Figure 7f, highlighting the considerable application potential of the FCI‐BT absorbing coating.

## 3 Conclusion

In conclusion, an NN‐based permittivity engineering strategy has been developed for the customizable design of magnetic microwave absorbers. The introduction of a “permeability locking‐permittivity optimization” paradigm effectively decouples the intricate interplay between electromagnetic parameters. Subsequently, a high‐throughput dual‐task screening framework coupled with an NN‐based model was established to accurately predict the permittivity requirements for both ideal and effective absorption, thereby transitioning the design methodology from empirical trial‐and‐error to a physics‐informed data fusion approach. This strategy was materialized in the synthesis of the FCI/BaTiO_3_ composites. The material experimentally validates the model's predictions by achieving a remarkable 5.1 GHz bandwidth at a mere 1.0 mm thickness, with an optimal *RL* of −45.12 dB attained at 5.16 GHz for a 1.9 mm thickness. Furthermore, the enhanced corrosion resistance demonstrates its excellent durability for practical applications. This AI‐guided framework not only provides a powerful tool for the rational design of magnetic absorbers but also establishes a generalizable paradigm extendable to other functional material systems, promising to significantly accelerate the discovery and deployment of advanced electromagnetic protection materials.

## 4 Experimental Section

### 4.1 Materials

Anhydrous ethanol (95%), glacial acetic acid, isopropanol, and n‐butanol were provided by Chengdu Haihong Chemical Co., Ltd. (China). Carbonyl iron powder (CIP) was purchased from BASF (Germany). Barium titanate (BaTiO_3_) particles with a size range of 0.6∼1.0 µm were obtained from Shandong Keyuan Biochemical Co., Ltd. (China). The silane coupling agents 3‐(Dimethylamino)propyltrimethoxysilane (C_26_H_58_ClNO_3_Si, DMAOP) and (3‐Glycidyloxypropyl)trimethoxysilane (C_9_H_20_O_5_Si, KH560) were supplied by Shanghai Macklin Biochemical Technology Co., Ltd. (China). Epoxy resin (DHH‐022) was sourced from Chengdu Dahao Paint Co., Ltd. (China).

### 4.2 Preparation of Flaky CIP

CIP was mixed with grinding media and anhydrous ethanol at a mass ratio of 1:20:10 in a ball‐milling jar. The mixture was ball‐milled for 8 h at rotational speeds of 200 rpm and 500 rpm, respectively. After milling, the resulting samples were collected and dried at 60°C for 6 h. The obtained products were designated as FCI‐200 and FCI‐500.

### 4.3 Preparation of FCI‐BT Coating

The FCI‐BT composite was dispersed in an epoxy resin at a mass fraction of 85%, and an appropriate amount of n‐butanol was added to facilitate mixing and dispersion, yielding the FCI‐BT coating. The resulting mixture was then uniformly sprayed onto a 180 mm × 180 mm metal substrate and dried in an oven for 8 h to form a coating suitable for subsequent bow test measurements.

### 4.4 Characterization

Field emission scanning electron microscopy (FE‐SEM) was performed using a ZEISS Sigma 300 microscope (Germany), and the elements were also analyzed by an energy dispersive spectrometer (EDS, OXFROD, X‐Max 80). Transmission electron microscopy (TEM) was conducted using an FEI Tecnai F20 microscope (USA) to analyze the composite microstructure. Fourier transform infrared (FTIR) spectra were obtained on a Nicolet 6700 (Thermo Fisher Scientific, Waltham, Massachusetts, USA) spectrometer. The structural properties of powder were explored through a wide‐angle X‐ray diffractometer (WAXRD, Bruker, PW1830, Philips) coupled with Cu Kα radiation (*λ* = 0.154 nm), and the incident angle for the measurement was 5∼80°. The elemental compositions were evaluated on an X‐ray photoelectron spectrometer (XPS, Thermo Sci‐entific K‐Alpha^+^) equipped with a monochromatic Al Kα X‐ray source (1486.6 eV). The Hysteresis loop test was measured by Vibrating Sample Magnetometer (LakeShore 8600). The electromagnetic parameters of the samples were measured using a vector network analyzer (VNA, Ceyear 3671D) across the 1∼18 GHz frequency range. The toroidal specimens (outer diameter: 7.00 mm; inner diameter: 3.04 mm) were fabricated by homogeneously mixing the sample powder with paraffin wax at a mass ratio of 85 wt.% for electromagnetic parameter characterization. The hydrophobicity was determined by a droplet shape analyzer (KRUSS, DSA25E). The electrochemical properties were measured by Shanghai Chenhua electrochemical workstation (CHI760E) with the conventional three‐electrode mode, including the working electrode (the coated Q235 steel with an exposed area of 1 cm^2^), the reference electrode (saturated silver chloride electrode), and the counter electrode (platinum sheet with 4 cm^2^ area). The electrochemical impedance spectroscopy (EIS) measurements were performed in the frequency range of 10^−2^–10^5^ Hz with the alternating current signal amplitude of 10 mV.

## Conflicts of Interest

The authors declare no conflicts of interest.

## Supporting information




**Supporting File**: advs74032‐sup‐0001‐SuppMat.docx.

## Data Availability

The data that support the findings of this study are available in the supplementary material of this article.
