# Supplementary material for: Neural Network‐Based Permittivity Engineering of Magnetic Absorbers for Customizable Microwave Absorption
Source: Adv Sci (Weinh). 2026 Jan 22;13(21):e21945. doi: 10.1002/advs.202521945 (PMC13073266; doi:10.1002/advs.202521945)
Supplement: Supplementary file 1 — Supporting File: advs74032‐sup‐0001‐SuppMat.docx. [file ADVS-13-e21945-s001.docx]

Supporting Information

**Neural Network-Based Permittivity Engineering of Magnetic Absorbers for Customizable Microwave Absorption**

Chenxi Liu^a, 1^, Jinzhe Li^b, 1, *^, Sen Li^c^, Zhongqiu Guo^a^, Yao Chen^d^, Tian Li^a^, Renchi Qin^a^, Jiaxu Sun^a^, Yongxi Lu^a^ and Fanbin Meng^a, *^

^a^ *Key Laboratory of Advanced Technologies of Materials (Ministry of Education), School of Materials Science and Engineering, Southwest Jiaotong University, Chengdu 610031, China*

^b^ *Shanghai Key Laboratory of Digital Manufacture for Thin-Walled Structure, Shanghai Jiao Tong University, Shanghai 200240, China*

^c^ *Shanghai Key Laboratory of Materials Laser Processing and Modification, School of Materials Science and Engineering, Shanghai Jiao Tong University, Shanghai 200240, China*

^d^ *Chongqing CEPREI Industrial Technology Research Institute Co., Ltd, Chongqing, 401332, China*

^1^ These authors contributed equally.

^*^ Corresponding author:

Jinzhe Li: [jinzheli22@sjtu.edu.cn](mailto:lima@swjtu.edu.cn), Fanbin Meng: [mengfanbin_wing@126.com](mailto:mengfanbin_wing@126.com).





**Figure S1.** Dataset overview for FCI-200 and FCI-500 under the dual-task screening strategy. The complete sets of results for **Task 1** and **Task 2** across all investigated thickness conditions.


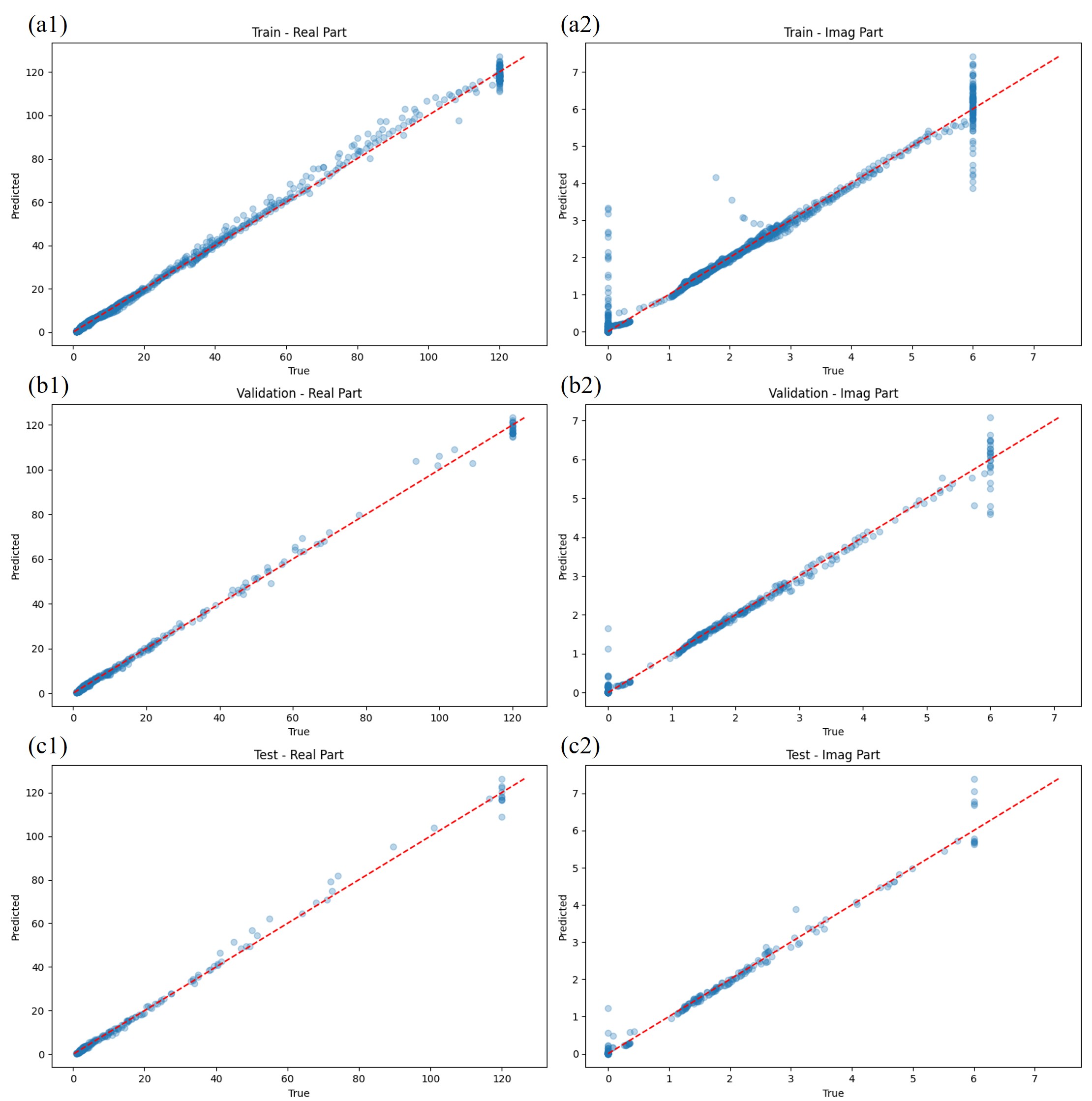


**Figure S2.** The predictive accuracy of the NN-based permittivity fitting model for FCI-200 under **Task 1**: Scatter plots of predicted values vs. true values for (a1-c1) *ε'* and (a2-c2) *ε''* across the training, validation, and test sets, respectively.





**Figure S3.** Comparison of the screening results and the NN-based permittivity fitting model predictions for *ε'*, *ε''* and the corresponding *RL* of FCI-200 under **Task 1**. The figure systematically presents the results for (a1-j1) *ε'*, (a2-j2) *ε''*, and (a3-j3) *RL* across a thickness range from 0.5 mm to 5.0 mm (in 0.5 mm increments).





**Figure S4.** Correlation of effective ε' boundaries with thickness and threshold for FCI-200 (**Task 2**). Screening of the upper and lower *ε'* boundaries of the effective region versus predictions from the NN-based permittivity fitting model. The results are presented for thresholds of (a1-e1) −10 dB, (a2-e2) −8 dB, and (a3-e3) −6 dB, each at thicknesses of 1.0 mm, 2.0 mm, 3.0 mm, 4.0 mm, and 5.0 mm.





**Figure S5.** Comparison of the screening results and the NN-based permittivity fitting model predictions for *ε'*, *ε''* and the corresponding *RL* of FCI-500 under **Task 1**. The figure systematically presents the results for (a1-j1) *ε'*, (a2-j2) *ε''*, and (a3-j3) *RL* across a thickness range from 0.5 mm to 5.0 mm (in 0.5 mm increments).





**Figure S6.** Correlation of effective *ε'* boundaries with thickness and threshold for FCI-500 (**Task 2**). Screening of the upper and lower *ε'* boundaries of the effective region versus predictions from the NN-based permittivity fitting model. The results are presented for thresholds of (a1-e1) −10 dB, (a2-e2) −8 dB, and (a3-e3) −6 dB, each at thicknesses of 1.0 mm, 2.0 mm, 3.0 mm, 4.0 mm, and 5.0 mm.


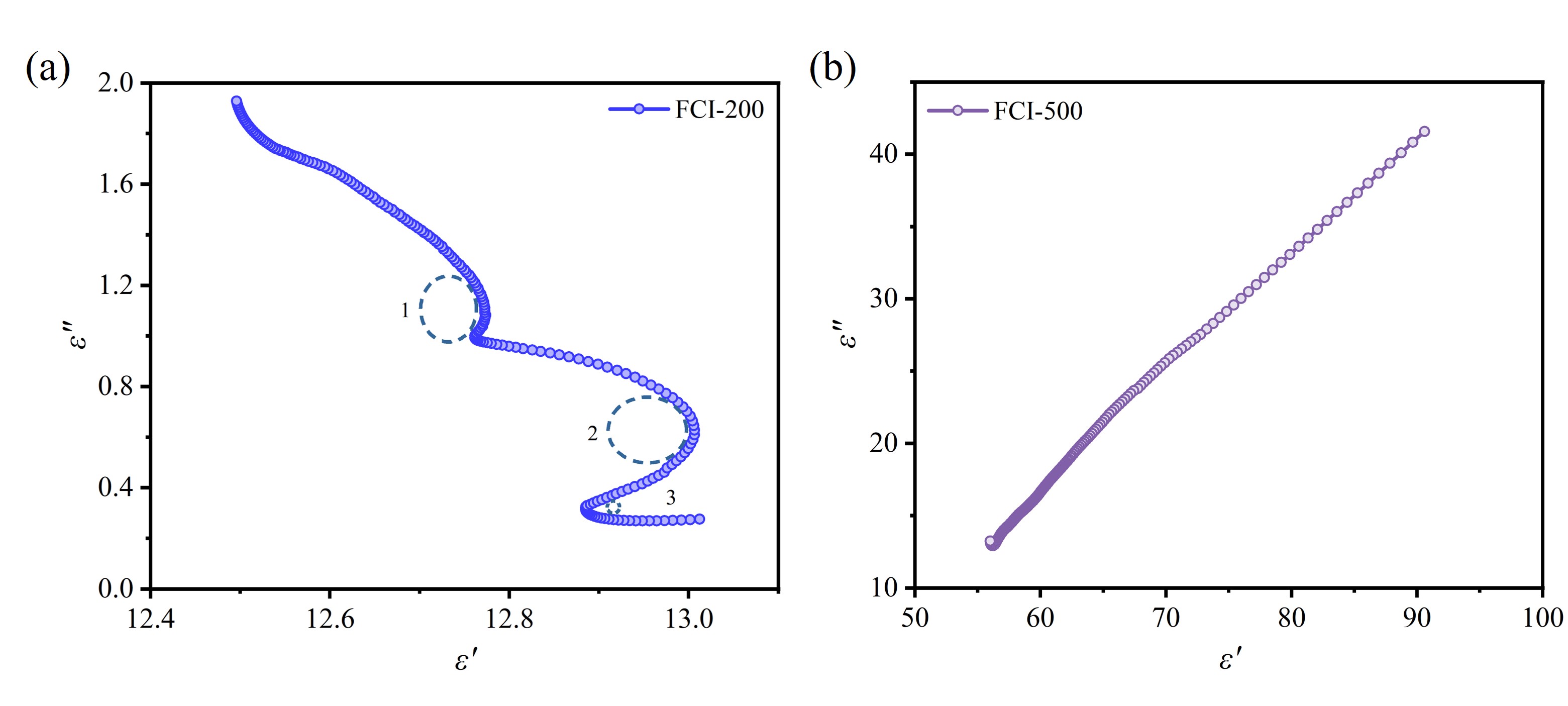


**Figure S7.** Cole-Cole plot of (a) FCI-200 and (b) FCI-500.


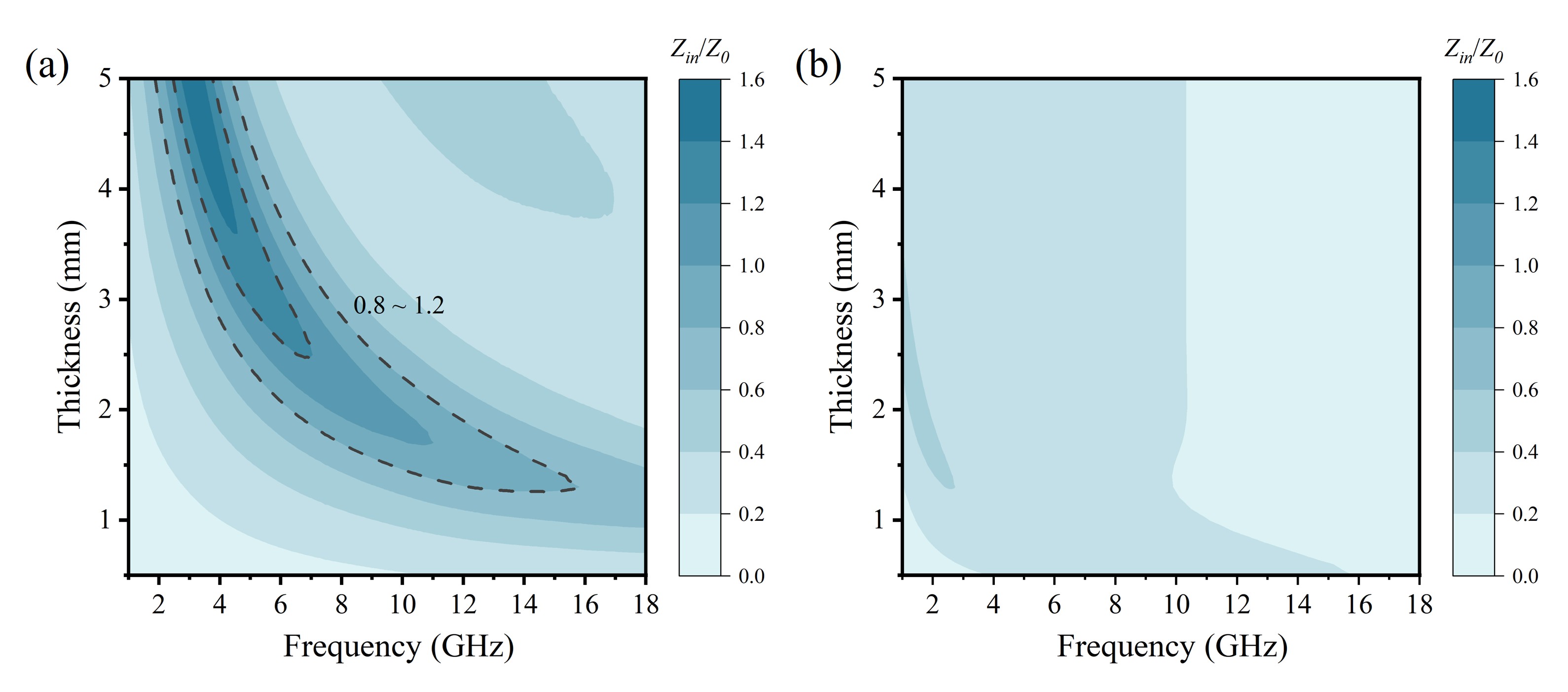


**Figure S8.** 2D mapping of |*Z_in_*/*Z_0_*| of (a) FCI-200 and (b) FCI-500.


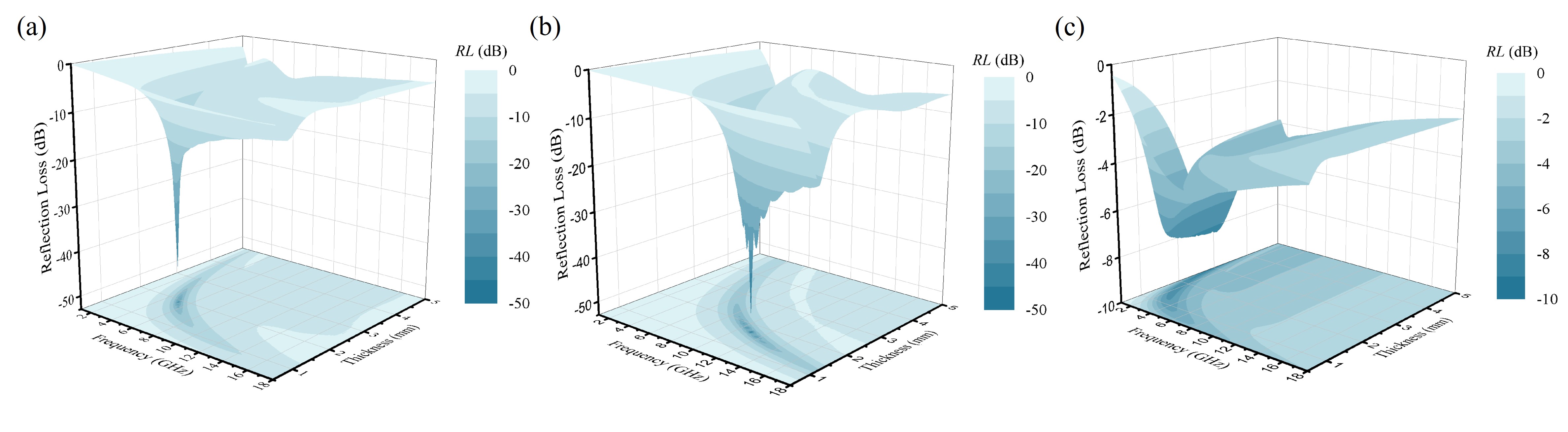


**Figure S9.** 3D *RL* of (a) FCI-BT, (b) FCI-200, and (c) FCI-500.


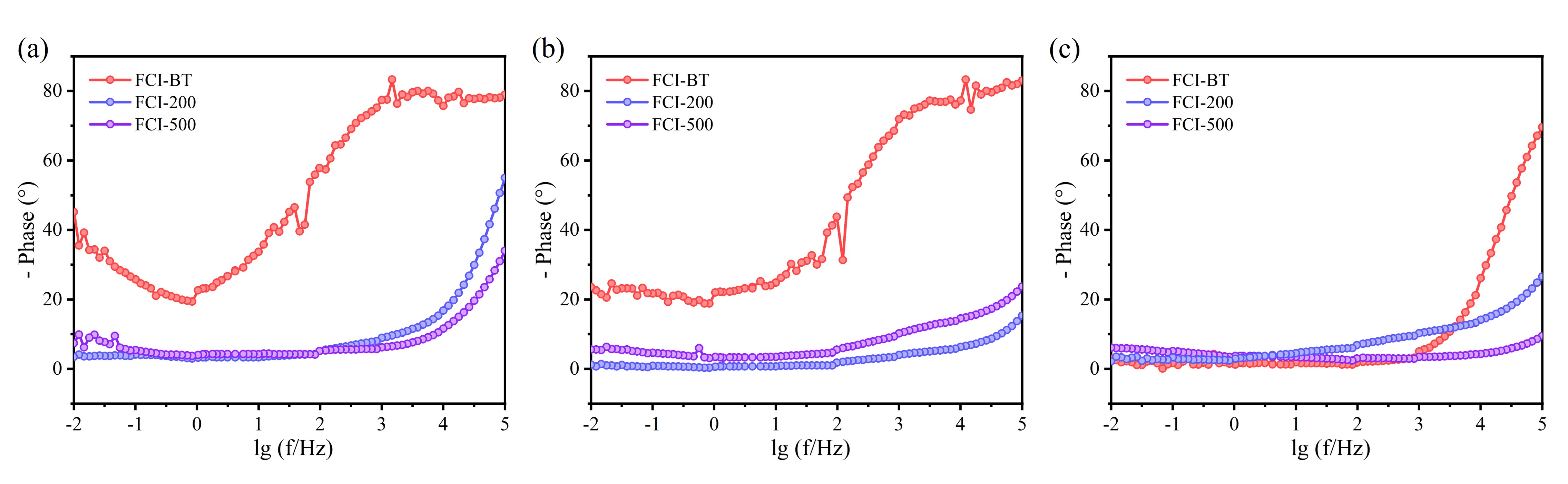


**Figure S10.** Bode phase plots of all samples after exposure to salt spray corrosion for (a) 3 days, (b) 5 days, and (c) 7 days.
